# Bacterial toxins induce non-canonical migracytosis to aggravate acute inflammation

**DOI:** 10.1038/s41421-024-00729-1

**Published:** 2024-11-05

**Authors:** Diyin Li, Qi Yang, Jianhua Luo, Yangyushuang Xu, Jingqing Li, Liang Tao

**Affiliations:** 1 College of Life Sciences, Zhejiang University, Hangzhou, Zhejiang China; 2 Research Center for Industries of the Future and Key Laboratory of Multi-omics in Infection and Immunity of Zhejiang Province, School of Medicine, School of Life Sciences, Westlake University, Hangzhou, Zhejiang China; 3 Center for Infectious Disease Research, Westlake Laboratory of Life Sciences and Biomedicine, Hangzhou, Zhejiang China; 4 Institute of Basic Medical Sciences, Westlake Institute for Advanced Study, Hangzhou, Zhejiang China

**Keywords:** Cell migration, Organelles

## Abstract

Migracytosis is a recently described cellular process that generates and releases membrane-bound pomegranate-like organelles called migrasomes. Migracytosis normally occurs during cell migration, participating in various intercellular biological functions. Here, we report a new type of migracytosis induced by small GTPase-targeting toxins. Unlike classic migracytosis, toxin-induced migrasome formation does not rely on cell migration and thus can occur in both mobile and immobile cells. Such non-canonical migracytosis allows the cells to promptly respond to microbial stimuli such as bacterial toxins and effectors and release informative cellular contents in bulk. We demonstrated that *C. difficile* TcdB3 induces liver endothelial cells and Kupffer cells to produce migrasomes in vivo. Moreover, the migracytosis-defective *Tspan9*^*‒/‒*^ mice show less acute inflammation and lower lethality rate in the toxin challenge assay. Therefore, we propose that the non-canonical migracytosis acts as a new mechanism for mammalian species to sense and exacerbate early immune response upon microbial infections.

## Introduction

Large clostridial toxins (LCTs) are a family of highly potent exotoxins produced by Clostridia, including *Clostridioides difficile* toxins TcdA and TcdB, *Clostridium perfringens* toxin TpeL, *Paeniclostridium sordellii* toxins TcsL and TcsH, and *Clostridium novyi* toxin Tcnα^1^. LCTs bind to varied cellular receptors and translocate their enzymatic domains through endosomes into the cytosol, where they inactivate members of Rho and Ras-family small GTPases by modifying the threonine residue in the GTP-binding pockets of GTPases^1–3^. The inactivation of small GTPases results in the disruption of actin cytoskeletons and cell body retraction^4^, which is commonly observed in intoxicated or infected cells. Early in the 1990s, researchers reported two divergent cell pathomorphological effects induced by LCTs: TcdA and prototypical TcdB (referred to as TcdB1) have an arborized appearance (D-type cytopathic effect) while TcsL and a variant TcdB (later referred as TcdB3)^5,6^ result in a spindle-like appearance (S-type cytopathic effect)^7,8^. The substrate preference of toxins was considered as the causal factor^9,10^, while cellular principles of these pathomorphological phenomena have never been closely analyzed^11–13^. LCTs such as *C. difficile* toxins are potent inducers of chemokines and cytokines^14,15^ and cause local and systematic acute inflammation in vivo^16,17^. How LCTs trigger acute inflammatory responses remains to be further elucidated; some studies suggested that Nlrp3 and Pyrin inflammasome-mediated innate immunity may contribute to these processes^18,19^.

Migrasomes are newly discovered membrane-bound organelles that mostly grow on the tips or intersections of the retraction fibers during cell migration^20^. These vesicular structures were first described in 2015, as large pomegranate-like vesicles in the extracellular spaces around normal rat kidney (NRK) cells^21^. Later studies showed that migrasomes exist on the retraction fibers of varied cell types in vitro and in vivo. Tetraspanins (TSPANs) are the most recognized proteins localized on migrasomes and retraction fibers (RFs) and contribute to migracytosis^22,23^. Depletion or overexpression of certain TSPANs, such as TSPAN1, TSPAN4, TSPAN7, and TSPAN9, strongly affects the migrasome-forming ability of the cells^24^. Recent studies also reported that the assembly of sphingomyelin synthase 2 (SMS2) initiates the migrasome formation^25^, and retraction fiber/microdomain-enriched TSPANs and phosphatidylinositol-bisphosphate (PIP2) drive the process^24,26–28^. Besides TSPANs, wheat germ agglutinin (WGA), a lectin that binds sialic acid and *N*-acetyl-*D*-glucosamine, is generally used to label migrasomes and RFs in many living cells^29^.

The process of migrasome forming, maturation, and release, which is called “migracytosis”, involves a series of biochemical reactions and molecular regulations^24,25,28,30,31^. Migracytosis may participate in various biological functions, including intercellular communication, mitochondrial quality control, redox signaling, organ morphogenesis, and angiogenesis^21,32,33^. During this process, cytoplasmic contents stored and released by migrasomes could be important for their functions. For example, it was shown that monocyte acts as a “vanguard” to provide a pro-angiogenic microenvironment through migrasomes depositing angiogenic factors, such as Cxcl12 and Vegfa^34^.

In this study, we report a non-canonical type of migracytosis induced by small GTPase-targeting toxins and effectors. Unlike canonical migracytosis, toxin-induced migrasome formation does not depend on active cell movement. In contrast, the non-canonical migracytosis could occur in both mobile and immobile cells and allow them to release informative cellular contents in bulk, as a prompt response to microbial stimuli such as bacterial toxins and effectors. We next demonstrated the toxin-induced migracytosis in the toxin-treated mice. Particularly, liver sinusoidal endothelial cells (LSECs) and Kupffer cells (KCs) are vulnerable to *C. difficile* TcdB3 and can form migracytosis both in vitro and in vivo. Lastly, we demonstrated that the migracytosis-defective *Tspan9*^*‒/‒*^ mice show much lower and delayed immune responses compared to the WT mice upon systematic exposure to TcdB3, suggesting that the non-canonical migracytosis is prominent to the toxin-induced sepsis.

## Results

### A *C. difficile* toxin induces migrasome formation in cultured cells

**Fig. 1:**
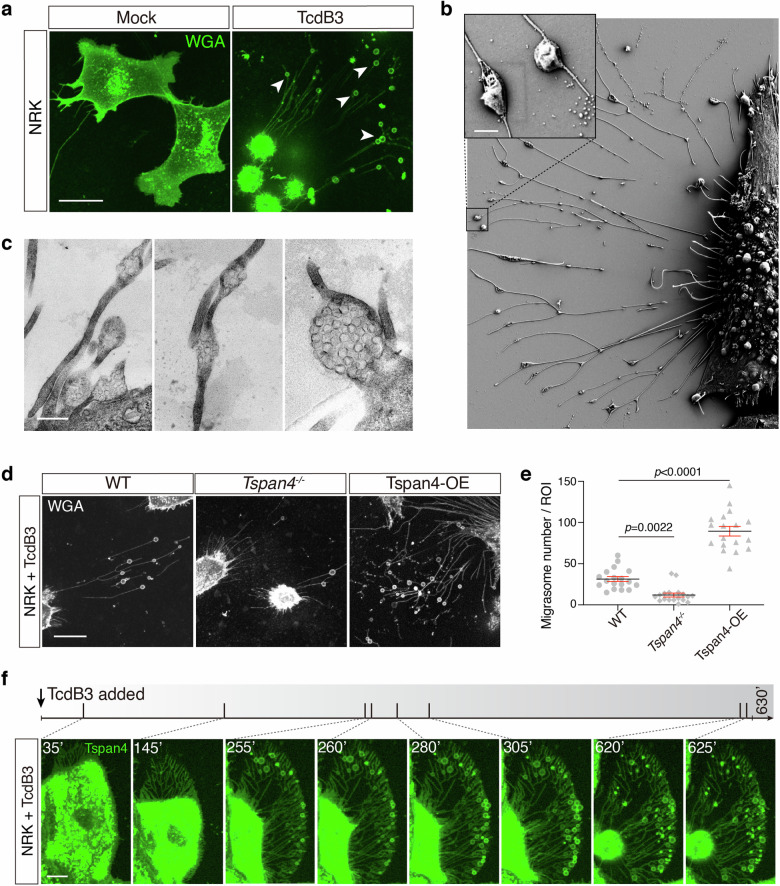
*C. difficile* TcdB3 induces migrasome formation in cultured cells. **a** Confocal image of the WT NRK cells staining by WGA treated with 0.2 pM TcdB3 or not for 4 h. Scale bar, 10 μm. **b** A representative SEM image of NRK cells treated with 0.2 pM TcdB3 for 4 h. An enlarged ROI was shown in the up-left corner. Scale bar, 500 nm. **c** Representative TEM images of NRK cells treated with 0.2 pM TcdB3 for 4 h. Scale bar, 500 nm. **d** Confocal images of the WT, TSPAN4 knockout, and TSPAN4-mCherry overexpressing NRK cells, which were treated with 0.2 pM TcdB3 for 4 h. The WT and TSPAN4 knockout cells were stained by WGA-AF488. Scale bar, 20 μm. **e** Quantification of migrasomes per field from **d**. Data are presented as mean ± SEM; *n* = 18. *P* values were calculated using ordinary one-way ANOVA followed by Dunnett’s test. **f** Time-lapse confocal imaging of NRK cells stably expressing TSPAN4-GFP treated with 0.2 pM TcdB3. The gray bar indicates the time scale of 630 min and the specific time points of each picture were labeled and marked as a short slash inside the gray time scale. Scale bar, 10 μm.

The subtype 3 *C. difficile* toxin B (TcdB3) is a TcdB variant that causes an S-type cytopathic effect, whereas subtype 1 TcdB (TcdB1) causes a D-type cytopathic effect. When applying TcdB3 to cultured cells, such as U2OS, SH-SY5Y, L929, Huh7, GES1, CAKi, and NRK cells, we serendipitously found that cells treated with TcdB3 produced massive hollow vesicles on thin filaments radiated from the retracted cell bodies (Fig. 1a; Supplementary Fig. S1). To further examine these toxin-induced filament-vesicle structures, scanning electron microscopy (SEM) and transmission electron microscopy (TEM) analysis were applied. The SEM result showed that multiple oval-shaped vesicles, ranging from 200 nm to 1 μm in diameter, connected to the thin filaments extended out from the retracted cell bodies (Fig. 1b). The TEM analysis further revealed that the oval-shaped vesicles contain a vast number of smaller particles (Fig. 1c). These morphological features well align with the description of prior reported migrasomes^21^.

Next, we managed to identify the molecular and biochemical characteristics of the vesicles by isolating them from the TcdB3-induced NRK cells. Under negative stain observation, the isolated vesicles showed ring-like structures attached to filaments, with an average diameter of ~500 nm. Liquid chromatography-mass spectrometry analysis for the vesicle-containing fraction showed the enrichment of many biomarkers reported for migrasomes^23,28^, including TSPAN family proteins, phosphatidylinositol glycan anchor biosynthesis class K (PigK), carboxypeptidase Q (CpQ), EGF domain-specific O-linked *N*-acetylglucosamine transferase (Eogt), and Rab35. Consistently, transiently expressed Tspan4-mCherry or Tspan7-GFP proteins largely overlapped with the toxin-induced vesicles in the NRK cells (Supplementary Fig. S2).

To further confirm that TcdB3 induces migracytosis, we recruited the NRK WT cells, *Tspan4*^*‒/‒*^ cells, and WT cells ectopically expressing a mouse Tspan4 protein (Tspan4-OE). These cells were treated with TcdB3 and their toxin-induced migracytosis were monitored. As expected, the Tspan4-OE cells produced more migrasomes than the WT cells, while the *Tspan4*^*‒/‒*^ cells showed the least number of migrasomes per cell and migrasome-forming cell percentage (Fig. 1d, e).

### Toxin-induced migracytosis is prompt and non-sustainable

To describe the dynamic process of TcdB-induced migracytosis, we applied real-time live-cell imaging and monitored the dynamic morphological changes of NRK cells stably expressing Tspan4-GFP upon exposure to TcdB3. Along with the cell matrix retraction, actin cytoskeleton-based structures such as peripheral membrane ruffling and filopodial extensions gradually disappear, leaving network structures resembling focal adhesion and retraction fibers. Ring-like structures occur on the retraction fibers, mainly on the distal side of cell retraction. These toxin-induced migrasomes would burst and release the ingredients, leaving dot-like remains. The life span of the migrasomes in the culture dish varied, from several minutes up to six hours (Fig. 1f; Supplementary Video S1).

There are several distinct features between canonical and non-canonical migracytosis. For canonical migracytosis, migrating cells continuously yield migrasomes at a relatively steady rate. In contrast, non-canonical migracytosis generates many migrasomes within a concentrated period, while no more migrasomes are formed afterward.

### Migracytosis is selectively induced by bacterial toxins and effectors

**Fig. 2:**
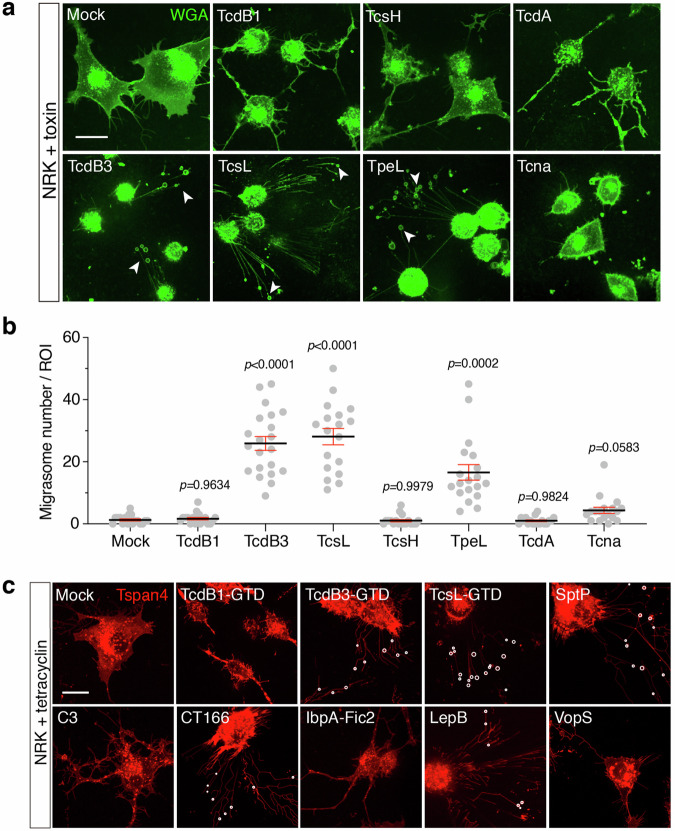
Migracytosis is selectively induced by bacterial toxins and effectors. **a** Representative confocal images of WGA-stained NRK cells treated with TcdB1 (0.2 pM), TcsH (3 nM), TcdA (1 nM), TcdB3 (0.2 pM), TcsL (1 nM), TpeL (30 nM) and Tcnα (3 nM) respectively for 3–4 h. White arrows indicate migrasomes. Scale bar, 20 μm. **b** Quantification of migrasomes per field from **a**. Data are presented as mean ± SEM; *n* = 18–21. *P* values were calculated using ordinary one-way ANOVA followed by Dunnett’s test. **c** Confocal images of NRK cells stably expressing TSPAN4-mCherry and inducible expressing different bacterial effectors respectively, including the enzymatic domains (GTD) from TcdB1, TcdB3, TcsL, and effectors including SptP, C3, CT166, IbpA-Fic2, LepB, and VopS. White circles represent actual migrasomes. Scale bar, 20 μm.

We next monitored whether other LCTs, including TcdB1, TcdA, TcsL, TcsH, TpeL, and Tcnα, induce migracytosis in the cultured cells. Interestingly, typical migrasomes can only be induced by some LCTs: TcsL and TpeL induced both the retraction fibers and migrasomes in a similar way to TcdB3. In contrast, the NRK cells treated with TcdB1, TcdA, and TcsH formed membrane-wrapped residues (irregular bubble-shaped) connected with thick bundles during the cell body retraction process. Cell retraction caused by Tcnα has several remnants outside the retracted cell bodies, but few protruded filaments were observed (Fig. 2a, b).

Because TcdB3, TcsL, and TpeL bind to very different cellular receptors^35–39^, we conjectured that the toxin-induced migrasome formation relies on the enzymatic activity but not the entry portal of toxins. To prove this, the enzymatic domains (glucosyltransferase domains, GTD) from TcdB3 and TcsL were expressed in the inducible form in the NRK Tspan4-mCherry cells. As anticipated, cytosolic expression of either GTD^TcdB3^ or GTD^TcsL^ caused migracytosis in the target cells (Fig. 2c). Besides, a TcdB3 mutant (D286N/D288N) lacking GTD activity did not show any cytopathic effect or toxin-induced migracytosis (Supplementary Fig. S3).

Many effector proteins and catalytic domains of toxins are structurally and functionally close^3^. Thus, we further tested certain bacterial effectors known to target Rho/Ras-family proteins, including SptP from *Salmonella enterica*^40^, C3 exoenzyme of *Clostridium limosum*^41,42^, CT166 from *Chlamydia trachomatis*^43^, IbpA-Fic2 from *Histophilus somni*^44^, LepB from *Legionella pneumophila*^45^, and VopS from *Vibrio parahaemolyticus*^46^. Notably, ectopic expression of certain effectors, such as SptP, CT166, and LepB, also induced migrasome formation (Fig. 2c).

### The activity of Rho is critical for migrasome formation

**Fig. 3:**
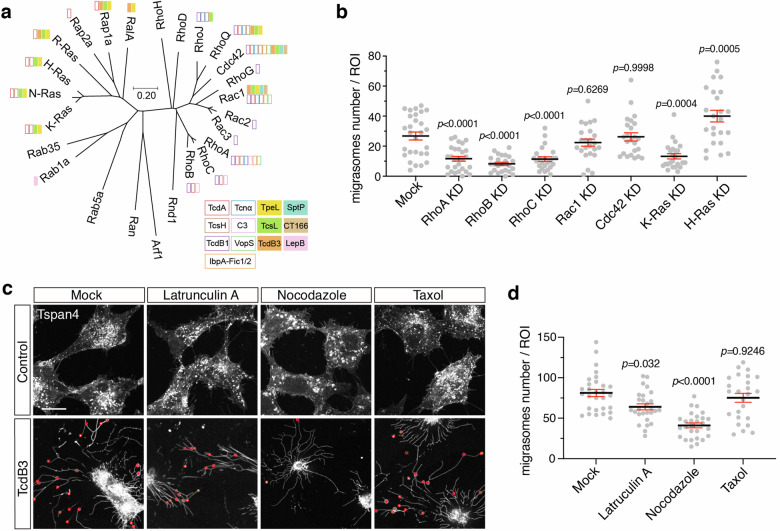
The activity of Rho is critical for migrasome formation. **a** The phylogenetic tree of mouse small GTPases. Amino acid sequences of 25 mouse small GTPases were aligned, and the phylogenetic tree was generated by MEGA software based on the Neighbor-Joining algorithm. Small GTPases that are targeted by specific toxins are labeled with colored boxes. The scale bar represents units of evolutionary distance. **b** Quantification of the numbers of migrasomes per field from multiple small GTPases knock-down cell lines. Data are presented as mean ± SEM; *n* = 24–28. *P* values were calculated using ordinary one-way ANOVA followed by Dunnett’s test. **c** Confocal images of NRK cells stably expressing TSPAN4-mCherry treated with cytoskeleton inhibitors and TcdB3 together. Cells were pretreated with Latrunculin A, nocodazole, and taxol for 30 min, and subsequently treated with 0.2 pM TcdB3 and inhibitors together for 3–4 h. Red circles indicate actual migrasomes. Scale bar, 20 μm. **d** Quantification of the numbers of migrasome from **c**. Data are presented as mean ± SEM; *n* = 25–29. *P* values were calculated using ordinary one-way ANOVA followed by Dunnett’s test.

Based on previous studies, the tested bacterial toxins and effectors prefer to target different members of Ras superfamily proteins^8,47–53^. Notably, TcdB3, TcsL, TpeL, SptP, CT166, and LpeB, which induce non-canonical migracytosis, inactivate varied small GTPases including Rac/Cdc42/Ras/Rab proteins, but always not RhoA/B/C (Fig. 3a). To investigate whether Rho GTPases also play central roles in regulating the canonical migracytosis, we knocked down Rho family GTPase members including RhoA, RhoB, RhoC, Rac1, and Cdc42 using the RNA interference experiment (Supplementary Fig. S4). Knocking down RhoA, RhoB, and RhoC drastically reduced number of migrasomes in the Tspan4-GFP NRK cells, whereas knocking down Rac1 or Cdc42 had little to no effect on migrasome formation. We also knocked down H-Ras, a Ras family member targeted by TcsL and TpeL. Cells with reduced H-Ras levels produced slightly more migrasomes, implying a complex regulatory network of small GTPases on migrasome formation (Fig. 3b; Supplementary Fig. S5a).

Recent studies showed the crosstalk of Rho GTPases in cell migration involving spatiotemporal dependent functions^54,55^. We verified the cellular localization of RhoA and Rac1 in the migrating NRK cells using live-cell imaging. Stronger RhoA signals were observed at the trailing edge of the cells. Intriguingly, RhoA is also enriched in the migrasomes. As a comparison, Rac1 largely resides in the leading edge of the migrating cells and is low in the migrasomes (Supplementary Fig. S5b). Considering the complexity of Rho GTPases-mediated regulation of cytoskeleton dynamics, we further recruited chemicals affecting cytoskeleton dynamics to explore the role of specific scaffolding components in migrasome formation. The NRK Tspan4-mCherry cells were pretreated with latrunculin A, nocodazole, or taxol, followed by exposure to TcdB3. Latrunculin A depolymerizes actin filaments, taxol stabilizes microtubules, and nocodazole depolymerizes microtubules. Pre-treatment with nocodazole inhibited the TcdB3-induced migrasome formation, while latrunculin A and taxol barely influenced the process (Fig. 3c, d). We also tested whether SMS2 and PIP2 contribute to the toxin-induced migracytosis in NRK cells using chemical inhibitors. Ly93 and SMS-IN-1, but not ISA-2011B, blocked TcdB3-induced migracytosis, suggesting that the activity of SMS2, but not PIP2, is also required for non-canonical migracytosis (Supplementary Fig. S6).

### Bacterial toxin induces migrasome formation in vivo

**Fig. 4:**
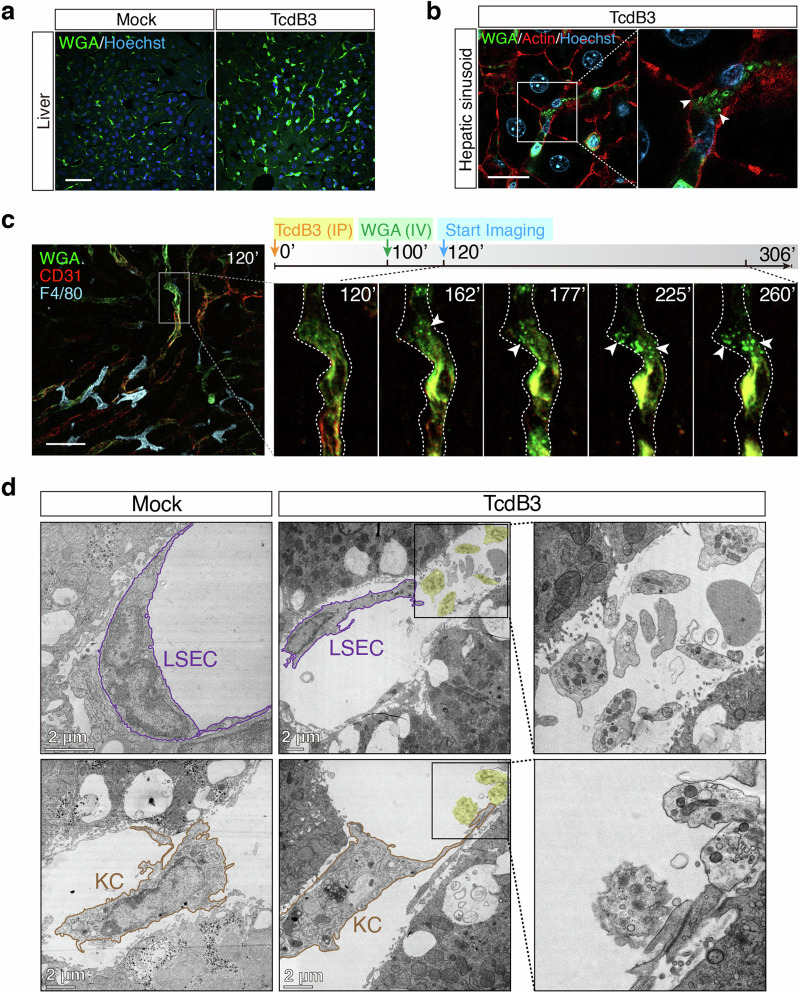
The bacterial toxin induces migrasome formation in vivo. **a** Liver sections of WT mice that IP injected with TcdB3 (0.4 μg/kg) or saline were stained by WGA-AF488 (green) and Hoechst (blue). Scale bar, 50 μm. **b** High-resolution confocal images of liver sections from TcdB3-treated mice. Arrowheads, migrasome-like structures around WGA^high^ signal cells in hepatic sinusoid. Scale bar, 20 μm. **c** Intravital imaging of mouse liver after IP injecting of TcdB3 over time. CD31-PE antibody labels LSECs (red), WGA-AF488 labels WGA^high^ cells (green), and F4/80-APC antibody (cyan) labels KCs in mouse liver. Specific time points after the TcdB3 injection were indicated in each image. Scale bar, 50 μm. **d** Representative TEM images of mouse hepatic sinusoid after IP injecting TcdB3 or not. The LSEC and KC were marked with outlines. Migrasome-like structures were highlighted with shaded labeling. Enlarged ROIs were arranged at right. Scale bars, 2 μm.

We next asked whether bacterial toxins can indeed induce migrasome formation in vivo. To answer this question, TcdB3 was chosen as a representative toxin and intraperitoneally (IP) injected into the mice (0.4 μg/kg). Such low toxin dosage is sufficient to cause a rapid immune response as shown by the surge of monocytes in the mouse blood (Supplementary Fig. S7). Four hours post-TcdB3 injection, mice were euthanized, and their hearts, livers, lungs, spleens, kidneys, and small intestines were dissected out, sectioned, and stained with WGA-AF488 (Supplementary Fig. S8). We noticed that the mouse liver sections showed particularly increased WGA signals upon TcdB3 injection (Fig. 4a), thus the liver was carefully monitored in the following experiments. Using super-resolution confocal microscopy, we further detected multiple oval-shaped vesicles alongside the cells in the hepatic sinusoids with diameters of ~1 μm (Fig. 4b), serving as strong evidence of migrasome formation in vivo.

To investigate the dynamic process of toxin-induced migracytosis in vivo, we have applied real-time live imaging for the mouse liver after the toxin injection. The live imaging revealed that the WGA-positive (WGA^+^) cells residing inside the hepatic blood vessels and sinusoids, mainly LSECs as labeled by CD31, had been lighting up by toxin stimulation over time, with migrasomes emerged at their rim region (Fig. 4c; Supplementary Video S2). Besides, the liver resident KCs, which are WGA-negative (WGA^‒^), may produce migrasomes upon TcdB3 induction as well (Supplementary Fig. S9a). We also isolated the primary endothelial cells and bone marrow-derived macrophages, which resembled LSECs and KCs, from the mice. These isolated cells are sensitive to TcdB3 and can easily form migrasomes upon toxin induction (Supplementary Fig. S9b, c).

To further validate that TcdB3 induces migracytosis of LSECs and KCs in vivo, the TEM analysis of mouse liver sections was performed. The TEM images revealed that the LSECs were damaged, shrunk, and shed off from the inner walls of hepatic sinusoids. Multiple migrasomes, with various small vesicles and mitochondrion inside, were observed adjacent to these damaged LSECs. Similarly, toxin-induced migrasomes were also found for KCs in the TEM images (Fig. 4d).

### Migracytosis-defective mice are resistant to TcdB3

**Fig. 5:**
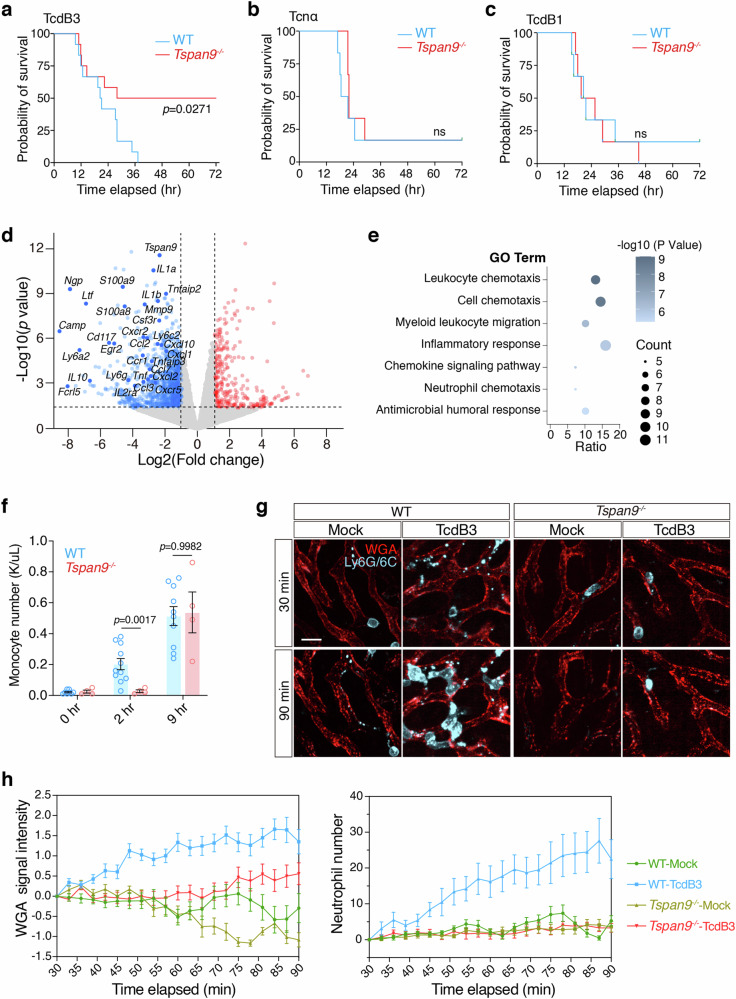
Migracytosis-defective mice are resistant to TcdB3. **a** Survival of WT and *Tspan9*^*‒/‒*^ mice after IP injection of TcdB3 (0.4 μg/kg) illustrated by the Kaplan–Meier curves (monitored for 3 days). **b** Survival of WT and *Tspan9*^*‒/‒*^ mice after IP injection of Tcnα (0.4 μg/kg) illustrated by the Kaplan–Meier curves (monitored for 3 days). **c** Survival of WT and *Tspan9*^*‒/‒*^ mice after IP injection of TcdB1 (0.4 μg/kg) illustrated by the Kaplan-Meier curves (monitored for 3 days). **d** Volcano plot illustrating differentially expressed genes in liver tissues of the *Tspan9*^*‒/‒*^ mice vs the WT mice under TcdB3 treatment. Liver tissues were collected 2 h after IP injecting TcdB3 (0.4 μg/kg). The plot highlights several representative genes significantly altered related to acute inflammation or chemokines, with a threshold set at |fold change (FC)| ≥ 2 and *P* value > 0.05. **e** The bubble plot illustrates the results of Gene Ontology (GO) enrichment analysis performed using the Metascape web tool for the gene set of significantly down-regulated genes in the *Tspan9*^*‒/‒*^ group. The analysis was conducted with a significance threshold set at *P* < 0.05. **f** Monocyte numbers in mouse whole blood were calculated 2 h or 9 h after TcdB3 IP injection. *n* = 4–10. The *P* values were calculated using two-way ANOVA followed by Bonferroni’s test. **g** Intravital imaging of mouse liver IP injected with TcdB3 (0.6 μg/kg) or not. WGA-AF488 labels liver sinusoids. Neutrophils are labeled with an anti-mouse Ly-6G/Ly-6C-PE antibody. Scale bar, 10 μm. **h** Quantification of WGA signal intensity and neutrophil number per field and per time-point from **g**. Data are presented as mean ± SEM and 10 fields from two mice are randomly selected in each group.

The next intriguing question is whether toxin-induced migracytosis has a physiological contribution to the disease progression or host response in vivo. We recruited the *Tspan9*^*‒/‒*^ mice which have been reported to have impaired migracytosis in many cells like macrophages and neutrophils^33^. Strikingly, ~50% of the *Tspan9*^*‒/‒*^ mice survived after the TcdB3 challenge (0.4 μg/kg TcdB3), while all the WT (*Tspan9*^*+/+*^) mice died within ~12–36 h (Fig. 5a). As a comparison, the WT and *Tspan9*^*‒/‒*^ mice were almost equally sensitive to Tcnα and TcdB1 (both do not induce migracytosis) in the toxin challenge assays (Fig. 5b, c).

### Toxin-induced migracytosis exacerbates early inflammation

As migrasomes are reported to contain various cellular ingredients for cell–cell communications^56,57^, we conjectured that toxin-induced non-canonical migracytosis also facilitates the bulk release of cellular ingredients as messengers in the early infection stage. In the cultured cells, we observed that TcdB3-induced migrasomes contain enriched signals for several cytokines and chemokines including Cxcl10, Ccl5, Vegfa, and IL-10 (Supplementary Fig. S10). Next, we examined the transcriptomes of livers in male mice at an early stage (~2 h) post-toxin exposure. The transcriptomic analysis of mouse livers revealed that the *Tspan9*^*‒/‒*^ mice have lower expression of many genes related to cell chemotaxis and inflammatory response, such as *Ly6a2*, *Cxcl10*, *Ccl2*, *Tnf*, and *Il1b*, compared to the WT mice (Fig. 5d, e; Supplementary Fig. S11 and Table S1). Consistently, the immunofluorescent analysis showed that TcdB3 induced overt expression of various chemokines and cytokines including IL-6, IL-1b, Vegfa, Cxcl12, Cxcl10, Ccl5, and Tnfα (Supplementary Fig. S12), indicating that the migracytosis-defective mice have a milder inflammatory response upon the stimulation of the bacterial toxin.

More interestingly, the WT mice showed a faster immune response to TcdB3 exposure compared to the *Tspan9*^*‒/‒*^ mice, as evaluated by the surge of plasma monocytes (Supplementary Fig. S13). This is particularly clear if compared to the male mice only (Fig. 5f). We further performed real-time live imaging for neutrophil infiltration in both the WT and *Tspan9*^*‒/‒*^ mouse livers. In the first 90 min post-IP injection of TcdB3, liver WGA signals increased faster and higher in the WT mice, compared to the *Tspan9*^*‒/‒*^ mice, indicating that fewer migrasomes were induced in the *Tspan9*^*‒/‒*^ mice. In line with the WGA signals, a massive number of neutrophils were attracted to the liver sinusoids within 90 min. Meanwhile, little to no neutrophil infiltration was detected in the livers of the *Tspan9*^*‒/‒*^ mice (Fig. 5g, h).

## Discussion

Migrasome is previously defined as an organelle produced during cell migration^21^. The cellular process of forming and releasing migrasomes, namely migracytosis, has been reported to mediate several biophysiological functions such as mitochondria quality control^33^, organ morphogenesis^32^, and angiogenesis^34^. Here, we demonstrate that bacterial toxins and effector proteins that target Rho and Ras family members induce migracytosis even in non-migrating cells, suggesting that migrasomes can be generated under certain conditions without active movement of cells. To separate it from the traditional way of migrasome formation, we define this toxin/effector-induced migracytosis as the non-canonical migracytosis.

It was reported that the assembly of sphingomyelin synthase 2 initiates the migrasome formation^25^ and retraction fiber/microdomain-enriched tetraspanins further drive the process^24,26^. However, little is known about the early signaling and regulation of migracytosis. We showed that the activity of Rho is critical to migrasome formation and RhoA is enriched in the migrasomes. Nocodazole, an inhibitor of microtubule formation, prohibits toxin-induced migracytosis. These results indicate that Rho signaling and local microtubule stability may involve the migrasome formation. In addition, we suggest that bacterial toxins and effectors targeting small GTPases could be used to investigate the biogenesis and regulation of migrasomes. Indeed, bacterial toxins historically serve as powerful tools to study many biological principles such as the control, regulation, and impact of signaling pathways in cells^12,58^.

Host regulatory GTPases, which play key roles in various cellular processes such as cell growth and differentiation, chemotaxis and motility, membrane dynamics, and vesicle trafficking, are vulnerable targets of microbial pathogens^3^. Many bacteria can produce exotoxins and effector proteins to modulate the activity of the GTPases and thus maneuver host cellular functions. Therefore, the non-canonical migracytosis may potentially allow the host to sense these pathogens and act as a mechanism to respond to microbial invasion. There is limited knowledge of how mammalian hosts sense small GTPase-targeting pathogens. The pyrin inflammasome is a previously characterized innate immune sensing machinery monitoring the inactivation of Rho proteins^19^. Albeit the exact sensing range remains to be established, migracytosis-mediated inflammation could be induced by the inactivation of Rac, Cdc42, and Ras. In this case, these two sensing mechanisms could complementarily conduct innate immune functions.

Bacterial toxins such as LCTs can inactivate Rho and Ras GTPases, leading to actin cytoskeleton disruption, causing cytopathic cell rounding, and eventually cell death^1,59,60^. Although it has been reported that high dose of LCTs may cause apoptotic and/or necrotic cell death^61–63^, intoxication with low concentration (low picomolar or ng/mL level) of LCTs was thought to lead to a slow cell death which takes days in vitro with no overt cell membrane rupture before the end^64,65^. However, the general perception that this cytopathic cell rounding is a “clean and quiet” process may not be accurate, as we showed that bacterial toxins and effectors can induce various cells to generate migrasomes, releasing a considerable amount of cellular ingredients including chemokines and cytokines.

On the other hand, systematic exposure to many LCTs at low doses (low μg/kg or ng/mL level) would effectively cause sepsis and kill mice within a couple of hours^5,66–69^. Using confocal live imaging and TEM, we further demonstrate that IP-injected TcdB3 induces migracytosis in the LSECs and KCs in vivo. We suggest that this migracytosis promotes the early release of inflammatory messengers, such as IL-6, Cxcl12, and Tnfα, and exacerbates acute inflammation. As evidence, we demonstrated that TcdB3 induces a massive infiltration of neutrophils in mouse livers following the induced LSECs migracytosis (indicated by the increasing WGA signals) in the WT mice, but not in the *Tspan9*^*‒/‒*^ mice. Consistently, higher blood monocyte levels were observed in the WT mice when compared to the *Tspan9*^*‒/‒*^ mice two hours post-toxin exposure. Strikingly, ~50% of the *Tspan9*^*‒/‒*^ mice survived in the toxin challenge assay. The *Tspan9*^*‒/‒*^ mice also showed lower immune response compared to the WT mice at early stages, indicating that toxin-mediated severe sepsis is largely prevented if migracytosis function gets impaired. In the current study, we mainly used TcdB3 for in vivo experiment and showed that this toxin largely induces migracytosis in the LSECs and KCs. Other toxins/effectors may target different cells and lead to varied manifestations. Future investigation into the role of migracytosis in the active infection by live bacteria could be crucial. In addition, we noticed a gender difference in TcdB3-induced migrasome-mediated inflammation in mice. The contribution of Tspan9 to migracytosis and other biological functions may vary between genders, while the underlying mechanism remains to be further investigated. Together, our findings renovate the current understanding of biogenesis and functions of migracytosis and provide new insights into how mammals rapidly sense and respond to microbial infections.

## Materials and methods

### Ethics statement

All animal procedures reported herein were performed following the institutional guidelines and approved by the Institutional Animal Care and Use Committee at Westlake University (IACUC Protocol #22-018-TL). To minimize the distress and pain, the mice injected with toxins were monitored every hour. Animals with signs of pain or distress such as labored breathing, inability to move after gentle stimulation, or disorientation were euthanized immediately. This method was approved by the IACUC and monitored by a qualified veterinarian.

### Mice

Specific pathogen-free (SPF) grade C57BL/6J mice (aged 6–8 weeks) were purchased from the Laboratory Animal Resources Center at Westlake University (Hangzhou, China). The C57BL/6J *Tspan9*^*‒/‒*^ mice were kindly gifted by Dr. Li Yu (Tsinghua University). All mice were supplied with food and water ad libitum and housed at 20–24 °C with 40–60% humidity and had a 12-h cycle of light/darkness (7 a.m. to 7 p.m.).

### Cell lines

The cell lines involved in this work include the normal kidney epithelial cell line NRK, mouse fibroblast cell line L929, human neuroblastoma cell line SH-SY5Y, human osteosarcoma cell line U2OS, human hepatoma cell line Huh7, human fetal stomach epithelium cell line GES-1, human renal cell carcinoma cell line CAKi and human embryonic kidney cell line 293T. All the cells were cultured in DMEM or RPMI-1640 medium supplemented with 10% fetal bovine serum (FBS) and 1% penicillin-streptomycin in a humidified atmosphere of 95% air and 5% CO_2_ at 37 °C. NRK and 293T cells were tested negative for mycoplasma contamination and authenticated via STR profiling (Shanghai Biowing Biotechnology Co. LTD., Shanghai, China).

### Reagents and antibodies

Reagents and antibodies were purchased from the commercial vendors: antibodies against IL-6 (A0286), Cxcl12 (A1325), Cxcl10 (A19138), Ccl5 (A14192), Vegfa (A12303), N-cadherin (A3045), IL-10 (A21701) were purchased from ABclonal Technology. Antibodies against CD31 (102407), F4/80 (123110), Ly6C (128010), Ly6G (127610), and TNF-α (506314) were from BioLegend. The antibody against IL-1b was from Biotechne (AF-401-NA). Alexa 488 (712-545-150), Alexa 594 (712-585-150), and Alexa 647 (111-605-003)-conjugated IgG antibodies were purchased from Jackson Immunoresearch. WGA-AF488 (W11261) was from Thermo Fisher. SIR-actin (CY-SC002) was generated by Cytoskeleton. Lipofectamine LTX Reagent and PLUS Reagent (15338100) were from Invitrogen Life Technologies (L3000015). Opti-prep (D1556) was from Sigma-Aldrich. Fibronectin (F0895) was from Sigma-Aldrich. Nocodazole (HY-13520), Taxol (HY-N0227), Puromycin (HY-K1057), Doxycycline (HY-N0565B), ISA-2011B (HY-16937), Ly93 (HY-114307), and SMS-IN-1 (HY-102041) were from MedChemExpress. Latruculin A (10010630) and Blasticidin S (14499) were from Cayman.

### Constructs

Genes encoding Tspan4-GFP or Tspan4-mCherry were cloned into the pLentiCas9 vector. Genes encoding full-length TcdB1, TcdB3, TcdA, Tcna, TcsL, TcsH, and TpeL were codon-optimized and synthesized by Genescript (Nanjing, China), and then cloned into pHT01 vector with His tag at the C-termini. Recombinant full-length TcdB1, TcdB3, TcdA, Tcnα, TcsL, TcsH, and TpeL proteins were expressed in *Bacillus subtilis* SL401 as described previously^5,66,67,70^.

DNA fragments encoding the GTD of TcdB1, TcdB3, and TcsL were subcloned into the pLVX-TetOn-GFP vector. DNA fragments encoding bacterial effectors including SptP, C3, CT166, IbpA-Fic2, LepB, and VopS were codon-optimized and synthesized by Genescript and cloned into pLVX-TetOn-GFP vector.

The selected shRNA sequences (rat *RhoA-1*: CCAAAGACGGAGTGAGAGA; rat *RhoA-2*: GGAAGAAACTGGTGATTGT; rat *RhoB-1*: GCAAGAAGCTGGTGGTGGT; rat *RhoB-2*: AGTGGGTGCCCGAGGTAAA; rat *RhoC-1*: GAAAGAAGCTGGTAATTGT; rat *RhoC-2*: CAGCAGGGCAAGAAGACTA; rat *Rac1-1*: TTGAGAAGCTGAAGGAGAA; rat *Rac1-2*: GTTAAGAAGAGGAAGAGAAAAT; rat *Cdc42-1*: GTTTGATGAAGCAATATTGGC; rat *Cdc42-2*: GCAATATTGGCTGCCTTGGAG; rat *H-Ras-1*: GAGTATGATCCCACTATAGAG; rat *H-Ras-2*: GGACTCCTACCGGAAACAGGT; rat *K-Ras-1*: GAGAGAGATCCGACAGTACAG; rat *K-Ras-2*: GAGATCCGACAGTACAGATTG;) were cloned into PLKO.1-TetOn vectors.

### Generation of small-GTPases knockdown cells

293T cells were used for lentivirus packaging. 293T cells were co-transfection of PLKO.1-TetOn plasmid containing each shRNA sequence, pSPAX2, and pMD2.G plasmids. After 48 h, the supernatant of the 293T culture was collected. The NRK-Tspan4-mcherry/GFP cells were transduced with Tet on-shRNA lentiviral vectors. After 24 h, the cells were supplied with fresh medium supplemented with 2.5 μg/mL Puromycin (HY-K1057, MedChemExpress) and cultured for another two days for selection.

### Generation of cells over-expressing bacteria effectors

For overexpressing the GTD of TcdB1, TcdB3, TcsL, NRK-Tspan4-mcherry cells were transduced with TetOn-effector-GFP lentivirus. After 6–8 h, the infected cells were imaged by a confocal microscope. For other mentioned effectors in this work, NRK-Tspan4-mcherry cells were transfected with pLVX-TetOn-GFP vector containing SptP, C3, CT166, IbpA-Fic2, LepB or VopS effector genes using the Lipofectamine LTX & PLUS Reagents (according to the protocol: http://tools.thermofisher.com/content/sfs/manuals/LipofectamineLTX_PLUS_Reag_protocol.pdf). The medium was changed to Doxycycline (HY-N0565B, MedChemExpress) containing medium (1 μg/mL) after 6 h and pictures were captured under the microscope after 12 h.

### Live-cell imaging and image analysis

Imaging of cell cultures was all conducted using a live-cell imaging system at 37 °C with 5% CO_2_. Glass-bottom plates (P24-1.5H-N, Cellvis) were coated with 10 μg/mL fibronectin (F0895, Sigma) dissolved in PBS for 1 h at 37 °C and washed with PBS three times before cell seeding. Cells were cultured for 10–12 h in fibronectin-precoated wells before imaging. Confocal images were acquired using a NIKON spinning-disk microscope (60*×* objectives) at 5280 *×* 5280 pixels per field. Images were processed with NIS-Elements analysis 5.0 and ImageJ. To quantify migrasome numbers in each group, a zone of 1700 *×* 1700 pixels was selected randomly, which contains 20–30 cells. Migrasomes were counted manually and statistically analyzed using GraphPad Prism 9.

### Induction of cytopathic cell-rounding effects

NRK cells were exposed to purified recombinant toxins and underwent similar cell-rounding progress. Concentrations for each toxin being used were as follows: 0.2 pM TcdB1, 0.2 pM TcdB3, 1 nM TcdA, 1 nM TcsL, 3 nM TcsH, 3 nM Tcnα, 30 nM TpeL and 100 pM TcdB3^D286N/D288N^. After 3–4 h of toxin treatment, most cells were undergoing the cytopathic cell-rounding process. Cells were stained by WGA-AF488 (0.5 μg/mL) for 10 min before imaging. For other cell types, including L929, SH-SY5Y, U2OS, Huh7, GES-1, and CAKi, the exposure time of TcdB3 slightly varied. Tspan4-GFP and Tspan4-mCherry stable cell lines did not need further WGA staining.

For TetOn-inducible expression cells, 10–12 h after seeding on plates coated with fibronectin, Doxycycline was added with a final concentration of 1 μg/mL to trigger the expression of bacterial effectors. After 8–12 h, live-cell imaging was performed to monitor the cytopathic effects.

### Isolation of toxin-induced migrasomes

NRK cells were cultivated in 150-mm dishes using a complete DMEM medium for 10 h, with a total of 30 dishes being prepared. Cells were treated with 0.2 pM TcdB3 for 4 h and harvested after trypsin digestion. Subsequent migrasome isolation steps were the same as described in the previous study^23^. In brief, while maintaining all procedures at 4 °C, cells were initially removed through centrifugation at 1000*×* *g* for 10 min. Subsequently, the removal of larger debris was accomplished by centrifugation at 4000*×* *g* for 20 min. In the third step, crude migrasomes were obtained as the pellet through centrifugation at 20,000*×* *g* for 30 min. This step was repeated after the pellet was washed with PBS. Next, utilizing Opti-prep (Sigma-Aldrich, D1556) as the density medium, migrasome fractionation was carried out through density gradient centrifugation. The step gradient in PBS, arranged from the lowest to the highest, was as follows: 30% Opti-prep, 25% Opti-prep, resuspended crude migrasome pellet in PBS, 15% Opti-prep, 12% Opti-prep, 10% Opti-prep, 8% Opti-prep, 5% Opti-prep, and 2% Opti-prep. Each step gradient contains 400 μL. Then the sample was subjected to density gradient centrifugation at 200,000*×* *g* for 208 min at 4 °C using an SW 60TI rotor (Beckman Coulter, Optima XPN-100). Following super-centrifugation, samples were systematically fractionated from top to bottom (384 μL per fraction). Each fraction was then mixed with an equal volume of PBS (384 μL) and centrifuged at 20,000*×* *g* for 30 min to collect the pellet. Pellets from different fractions were washed with PBS, divided into 3 sub-fractional packs, and further centrifuged at 20,000*×* *g* for 30 min. The final collected pellet was utilized for negative stain observation and proteomics analysis.

### SEM

NRK cells were cultured on fibronectin-precoated coverslips in a complete DMEM medium for 10 h and subsequently treated with 0.2 pM Tcdb3 for 4 h. Following this, cells were fixed in 2.5% glutaraldehyde in 0.1 M PB buffer for 2 h at room temperature, washed 3 times with ddH_2_O, and post-fixed with 2% Auq OsO_4_ containing 1.5% potassium ferrocyanide for 60 min at room temperature. After being washed 3 times with ddH_2_O, samples were dehydrated using an ethanol series (50%, 70%, 90%, 95%, and twice in 100%) for 8 min each. The specimens were subjected to critical point drying (Leica-EM CPD300, Germany) with carbon dioxide as the transitional fluid. The heating process was conducted in 1 °C /min steps until reaching the critical point (31 °C and 74 bar). Subsequently, the dried samples were sputter-coated with an ~5–10 nm-thick gold film before examination using a field emission scanning electron microscope with an SE detector at an acceleration voltage of 3 kV.

### TEM

#### Cultured cell TEM

For morphological analysis of cultured cells, samples were cultivated in 35 mm dishes and fixed with a mixture of 2.5% glutaraldehyde and 2% PFA in 0.1 M PB buffer for 30 min at room temperature. After fixation, samples were washed three times with PB buffer and post-fixed with 1% osmium containing 1.5% potassium ferrocyanide for 60 min. Subsequently, samples underwent dehydration with a graded series of ethanol (50%, 70%, 90%, 95%, and twice in 100%) for 5 min each. Finally, samples were infiltrated with and embedded in EPON12 resin. After polymerization for 48 h at 60 °C, 70 nm-thick ultrathin sections were cut using a diamond knife and subsequently picked up with Formvar-coated copper grids (100 mesh). The sections were post-stained with uranyl acetate and Sato’s Lead. The samples were then examined using a Thermo Fisher Talos 120 transmission electron microscope.

#### Negative staining of isolated migrasome

Purified migrasome or exosome pellets were resuspended in 50–100 μL PBS, then a 5 μL sample of each was mixed with an equal volume of 2.5% glutaraldehyde (PB, pH 7.4) and fixed for 30 min at room temperature. The sample was spread onto glow-discharged Formvar-coated copper mesh grids (Electron Microscopy Sciences, Hatfield) for about 5 min, then washed with water before staining with uranyl acetate for 2 min. After blotting off the excess staining solution with filter paper, the copper mesh grids were washed with water. Post-drying, grids were imaged at 80 kV using a transmission electron microscope ThermoFisher Talos 120.

#### Liver tissue TEM

At 6–8 weeks old, male WT mice received IP injections of TcdB3 (0.4 μg/kg) or saline. After 4 h, mice were anesthetized by IP injection of 1% pentobarbital sodium. Transcardial perfusion was performed with sterile ice-cold 1*×* PBS for 10 min at a rate of 7.5 mL/min to euthanize the mice and clear their vasculature of blood. Perfusion was continued with ice-cold 4% paraformaldehyde for an additional 10 min at the same rate. Exposed viscera were kept wet with sterile 1*×* PBS and covered with a small bag of ice during perfusion. Following perfusion, liver tissue was promptly sampled and placed in 2.5% glutaraldehyde and 2% PFA in 0.1 M PB buffer at 4 °C overnight. After fixation, samples were washed three times with PB buffer and post-fixed with 2% osmium for 60 min on ice, followed by fixation with 2% osmium containing 2.5% potassium ferrocyanide for an additional 60 min on ice. Samples were then dehydrated with a graded series of ethanol (30%, 50%, 70%, 85%, 95%, and twice in 100%) for 10 min each. Subsequently, samples were infiltrated with and embedded in EPON12 resin. After ultrathin sectioning and post-staining, the samples were imaged using Thermo Fisher Talos 120.

### Functional inhibitory assay

NRK cells stably overexpressing Tspan4-mCherry were cultured on glass-bottom plates precoated with fibronectin for 10–12 h. Subsequently, the cells were incubated with complete DMEM supplemented with 10 nM Latruculin A (Cayman, 10010630), 1 μM Nocodazole (MedChemExpress, HY-13520), 1 μM Taxol (MedChemExpress, HY-N0227), 20 μM ISA-2011B (MedChemExpress, HY-16937), 35 μM Ly93 (MedChemExpress, HY-114307), or 30 μM SMS2-IN-1 (MedChemExpress, HY-102041) for 30 min, respectively. Following the incubation, the cells were treated with 0.2 pM TcdB3 for 4 h before imaging.

### Toxin challenge assay

WT or *Tspan9*^*‒/‒*^ mice (balanced between males and females), aged 6–8 weeks, were intravenously injected with TcdB3 (0.4 μg/kg), TcdB1 (0.4 μg/kg), or Tcnα (50 μg/kg). The time of death for each mouse was then recorded.

### Intravital imaging of mouse liver

As reported previously^33^, intravital imaging was conducted using an inverted spinning-disk confocal microscopy system (Dragonfly 200, Andor) to observe dynamic changes in mouse liver tissue following TcdB3 induction. One hour after an IP injection of TcdB3 (0.6 μg/kg) or saline, WT or *Tspan9*^*‒/‒*^ male mice, aged 8–12 weeks, were intravenously injected with 3 μg of WGA-AF488 (Thermo Fisher, W11261), 3 μg of PE-CD31 (Biolegend, 102407), and 3 μg of APC-F4/80 (Biolegend, 123110) or APC-Ly-6G (Biolegend, 127610)/Ly-6C (Biolegend, 128010) antibodies. Five minutes later, anesthesia was induced in the mice via IP injection of Avertin (375 mg/kg). The mice were then dissected to expose the liver and positioned on a circular glass slide for time-lapse imaging. Images were processed using Imaris 9.3 and ImageJ software, with some images being deconvoluted using Huygens professional software for better presentation. The WGA signal intensity was quantified using ImageJ, and the neutrophil count was quantified using Imaris 9.3.

### Immunofluorescence

To investigate the in vivo surge of WGA^High^ signal following bacterial toxin exposure, tissues from the liver, lung, kidney, spleen, heart, and small intestine from the WT or *Tspan9*^*‒/‒*^ mice (male, 6–8 weeks) were promptly collected upon euthanasia. The tissues were fixed in freshly prepared 4% paraformaldehyde at 4 °C overnight with gentle rotation and then washed with 1*×* PBS to remove excess paraformaldehyde. Samples underwent dehydration in a PBS buffer containing 30% sucrose at 4 °C overnight with gentle rocking until tissues sank. After removing excess sucrose, samples were embedded in an OCT compound, rapidly frozen, and sectioned into thin slices (10 μm) using a cryostat (Lecia-CM1950, Germany). Sections were permeabilized for 5 min with 0.1% Triton X-100 in 1*×* PBS (PBST), followed by staining with WGA-AF488 (1:500) and Hoechst (1:1000) for 30 min at room temperature in the dark. Subsequently, sections were washed three times with 1*×* PBST and visualized using a NIKON spinning-disk microscope (40*×* objective) or a ZEISS LSM900 confocal microscope (63*×* objective with an Airyscan detector).

For immunofluorescent detection of cytokines, chemokines, or cell markers in the liver, frozen liver tissue sections were prepared as described previously^28^. After permeabilization in 1*×* PBST, sections were blocked with 5% donkey serum (Jackson ImmunoResearch, 017-000-121) in 1*×* PBST for 1 h at room temperature. The sections were incubated with antibodies against N-cadherin, IL-6, Cxcl12, Ccl5, TNF-α, Cxcl10, VEGFA, IL-1b (1:100) at 4 °C overnight. Then the slides were washed three times with 1*×* PBST for 10 min each. Next, the sections were incubated with Alexa 561 or Alexa 647-conjugated IgG antibody (1:100) for 1 h at room temperature in the dark. Following this, the sections were washed and stained by WGA-AF488 (1:500) and Hoechst (1:1000). Finally, after washing with PBST, the sections were imaged using an OLYMPUS spinning disk confocal microscope (40*×* objective) at 2304 *×* 2304 pixels per field. Image processing was performed with CellSens Dimension 4.0 and ImageJ software.

For immunofluorescent detection of cytokines and chemokines in the toxin-induced L929 cells, samples were prepared as previously described. Briefly, cells were fixed with a solution consisting of a 1:1 mixture of cell culture medium and 4% paraformaldehyde in PBS (Beyotime, P0099) for 5 min. These cells were then fixed with 4% paraformaldehyde in PBS for another 5 min, permeabilized with PBS containing 0.05% saponin and 10% FBS for 30 min, stained with respective antibodies following the manufacturer’s instruction, and subjected to confocal imaging.

### Mouse blood collection

In this study, blood collection from mice was performed via cardiac puncture. WT or *Tspan9*^*‒/‒*^ male mice, aged 6–8 weeks, were anesthetized with an IP injection of 1% pentobarbital sodium at specific time points following treatment with either TcdB3 (0.4 μg/kg) or saline. After anesthesia, the hearts were exposed, and blood was carefully withdrawn from the left ventricle using a syringe. Special care was taken to avoid contamination and minimize stress to the animals during the procedure. The collected blood samples were then subjected to routine tests to analyze various hematological parameters, including red blood cell count, white blood cell count, hemoglobin levels, and platelet count.

### RNA-seq analysis

WT or *Tspan9*^*‒/‒*^ mice (male, 6–8 weeks) were sacrificed 2 h after IP injection of TcdB3 (0.4 μg/kg) or saline, and freshly collected liver tissue sections (50–100 mg) were flash-frozen and stored in liquid nitrogen. Following RNA purification, reverse transcription, and library construction, sequencing was performed at Shanghai Majorbio Bio-pharm Biotechnology Co., Ltd. (Shanghai, China). Conducting differential expression analysis involves assessing quantitative expression results to identify genetic variations between groups. Differential expression is determined by evaluating the gain of differentially expressed genes between two groups using the variance analysis software EdgeR. The applied threshold criteria for significance are set as |log_2_FC| ≥ 1 and *P* value < 0.05.

### Real-time qPCR

To determine the knock-down efficiencies of RhoA, RhoB, RhoC, Rac1, Cdc42, HRas, and KRas in NRK cells, WT and knock-down cell lines were seeded on plates at 25% confluence and cultured for 10–12 h. Doxycycline was added to a final concentration of 1 μg/mL to trigger the generation of shRNA for 36 h. Whole-cell extracts were isolated using a MolPure Cell/Tissue Total RNA Kit (Yeasen, 19221ES50). RNA reverse transcription was performed using ABScript III RT Master Mix for qPCR with gDNA Remover (ABclonal, RK20429) from 2 μg of total RNA. qPCR experiments were carried out with universal SYBR Green Fast qPCR Mix (ABclonal, RK21203) using Biorad CFX Connect Real-Time PCR Detection System (Biorad, USA). The real-time PCR of *RhoA* (*RhoA*-F: ATGAGCACACAAGGCGGGAG, *RhoA*-R: ACAAGATGAGGCACCCCGAC), *RhoB* (*RhoB*-F: AAGTGTACGTGCCCACCGTG, *RhoB*-R: GATGACGTCGGTGTCCGGGT), *RhoC* (*RhoC*-F: ACCCGGACACTGACGTCATC, *RhoC*-R: GCTCATCTTGCCTCAGGTCCT), *Rac1* (*Rac1*-F: CTCTGGGACACAGCTGGACA, *Rac1*-R: CGATCTCTTTCGCCATGGCT), *Cdc42* (*Cdc42*-F: TATGTGGAGTGTTCCGCCCT, *Cdc42*-R: AGCTGTGCAGAAAGGGCTCT), *H-Ras* (*H-Ras*-F: GTGGGAAAGAGTGCCCTGAC, *H-Ras*-R: CCTGTGCGCATGTACTGGTC), *K-Ras* (*K-Ras*-F: GTAGTTGGAGCTGGTGGCGT, *K-Ras*-R: CCTCTTGACCTGCTGTGTCGAG) were performed and the relative gene expression was calculated by normalizing to *Gapdh* (*GAPDH*-F: GTATCGGACGCCTGGTTACC, *GAPDH*-R: ATACTCAGCACCAGCATCAC) mRNA.

### Statistical analysis

For the analysis of in vitro experiments, ordinary one-way ANOVA followed by Dunnett’s test was used to determine the statistical differences between the control and experimental groups. For in vivo analysis, the Mann–Whitney test was employed to assess statistical differences between two groups, and multiple comparisons were conducted using two-way ANOVA followed by Bonferroni’s test. All tests were two-tailed, and a *P* value of < 0.05 was considered statistically significant. Data were plotted and analyzed using GraphPad Prism (version 9.0) and represented as mean ± SEM or mean ± SD. Detailed statistical information, including the tests used and the exact value of *n*, is provided in the figure legends. Here, *n* denotes the exact number of animals for in vivo studies and the exact number of biological repeats for in vitro studies.

## Supplementary information

Supplementary Information

Supplementary Video S1

Supplementary Video S2

Supplementary Table S1

## Data Availability

The data that support the findings of this study are available from the corresponding author upon reasonable request. Source data are provided in the manuscript.
