# Supplementary material for: Bacterial toxins induce non-canonical migracytosis to aggravate acute inflammation
Source: Cell Discov. 2024 Nov 5;10:112. doi: 10.1038/s41421-024-00729-1 (PMC11538519; doi:10.1038/s41421-024-00729-1)
Supplement: Supplementary file 1 — Supplementary Information [file 41421_2024_729_MOESM1_ESM.pdf]

Supplementary Information for

Bacterial toxins induce non-canonical migracytosis to aggravate acute inflammation

Diyin Li[1,2,3,#], Qi Yang[1,2,3,#], Jianhua Luo[2,3,4], Yangyushuang Xu[2,3], Jingqing Li[2,3], Liang Tao[1,2,3,4,*]

[1] College of Life Sciences, Zhejiang University, 310058, Hangzhou, Zhejiang, China

[2] Research Center for Industries of the Future and Key Laboratory of Multi-omics in Infection and Immunity of Zhejiang Province, School of Medicine, School of Life Sciences, Westlake University, 310030, Hangzhou, Zhejiang, China

[3] Center for Infectious Disease Research, Westlake Laboratory of Life Sciences and Biomedicine, 310024, Hangzhou, Zhejiang, China

[4] Institute of Basic Medical Sciences, Westlake Institute for Advanced Study, 310024, Hangzhou, Zhejiang, China

[#] These authors contributed equally to this work.

* Corresponding to:

Liang Tao, Ph.D. Email: taoliang@westlake.edu.cn

Supplementary Figures

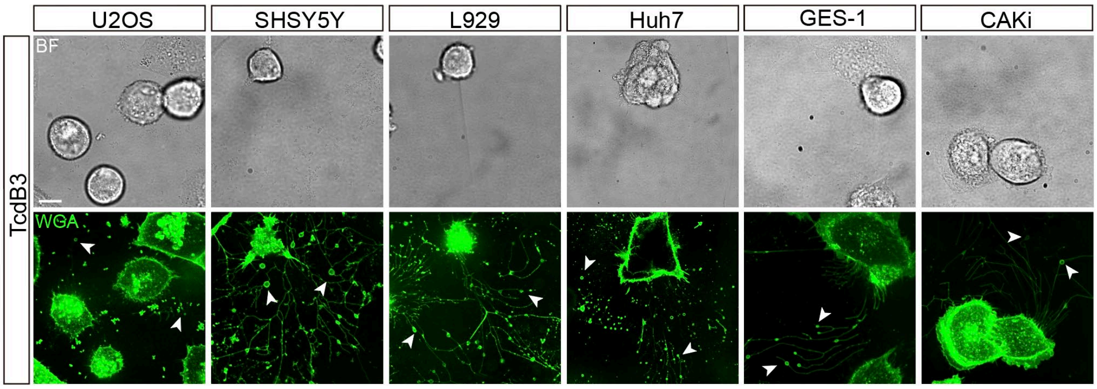

Supplementary Fig. S1 Bright-field and confocal images of multiple wild-type cell lines, including U2OS, SH-SY5Y, L929, Huh7, GES-1, and CAKi, staining by WGA treated with TcdB3 or not. Scale bar, 10 μm.

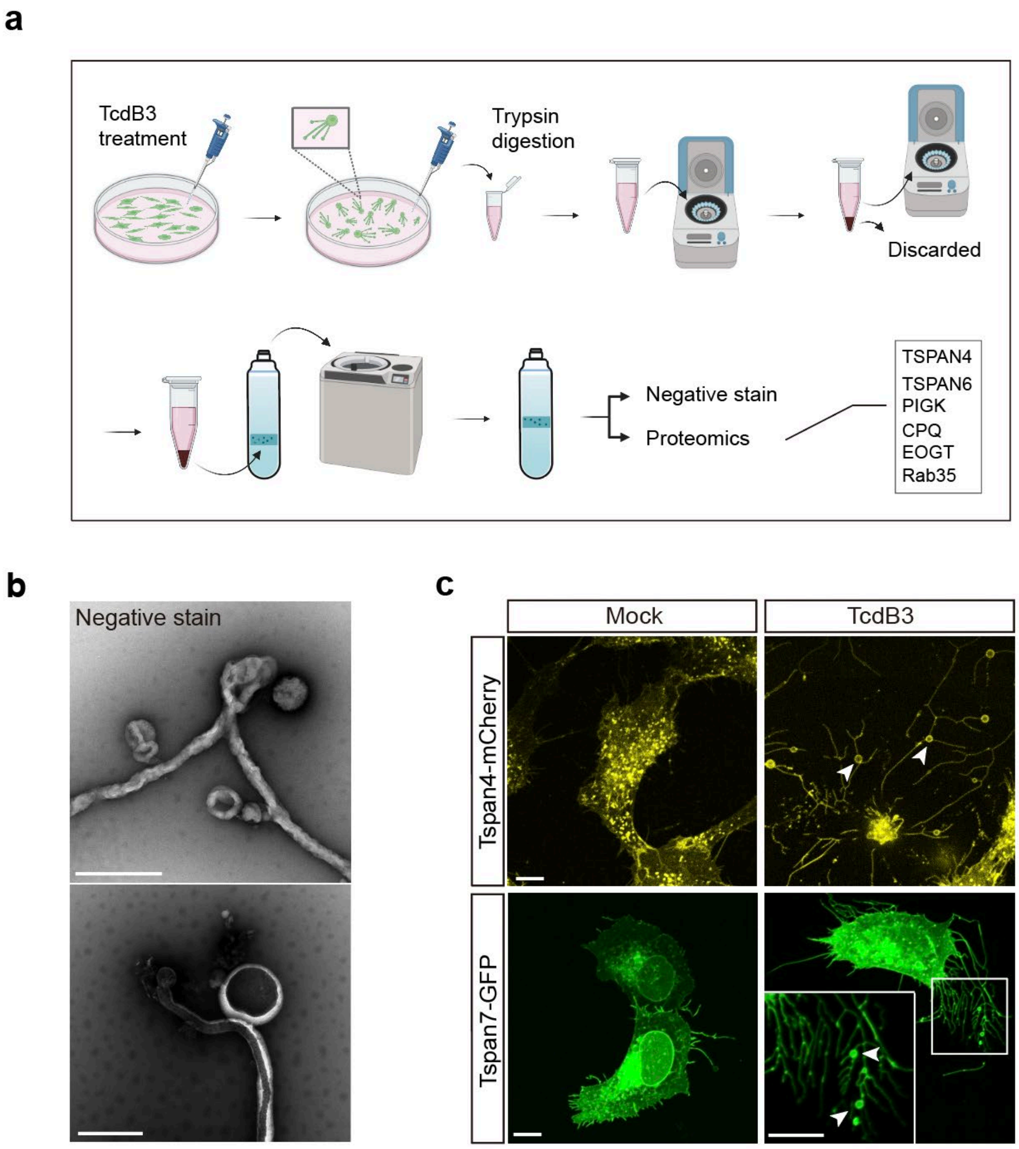

Supplementary Fig. S2 Further identification of the TcdB3-induced migrasome. a Schematic view of migrasome purification after TcdB3 treatment. Marker proteins of migrasome were identified from the purified sample by mass spectrum analyses. **b** Representative TEM images of negatively stained samples of migrasomes purified from TcdB3-induced NRK cells overexpressing TSPAN4-GFP. Scale bar, 500 nm. **c** Confocal images of NRK cells stably overexpressing TSPAN4-mCherry or TSPAN7-GFP treated with 0.2 pM TcdB3 or not. Scale bar, 10 μm.

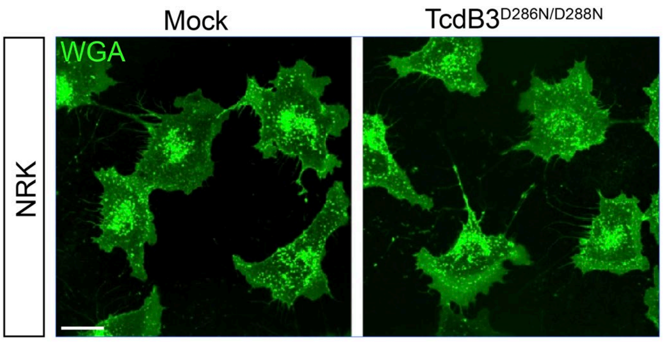

Supplementary Fig. S3 Confocal images of wild-type NRK cells staining by WGA treated with TcdB3$^{D286N/D288N}$. Scale bar, 20 µm.

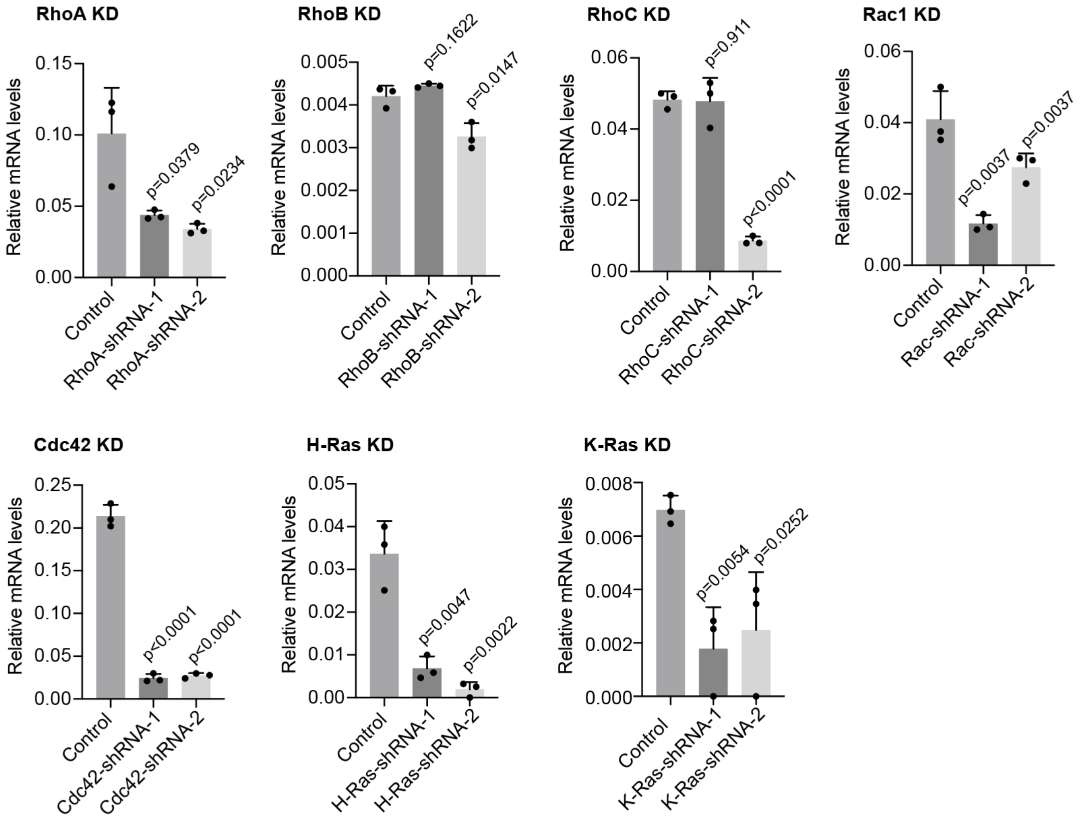

Supplementary Fig. S4 Relative expression levels of small GTPases mentioned in this work in response to inducible knocking down. Results are presented as fold change compared to the control group. Error bars represent standard deviation (SD) from triplicate experiments. *p* values were calculated using a two-tailed, unpaired t-test.

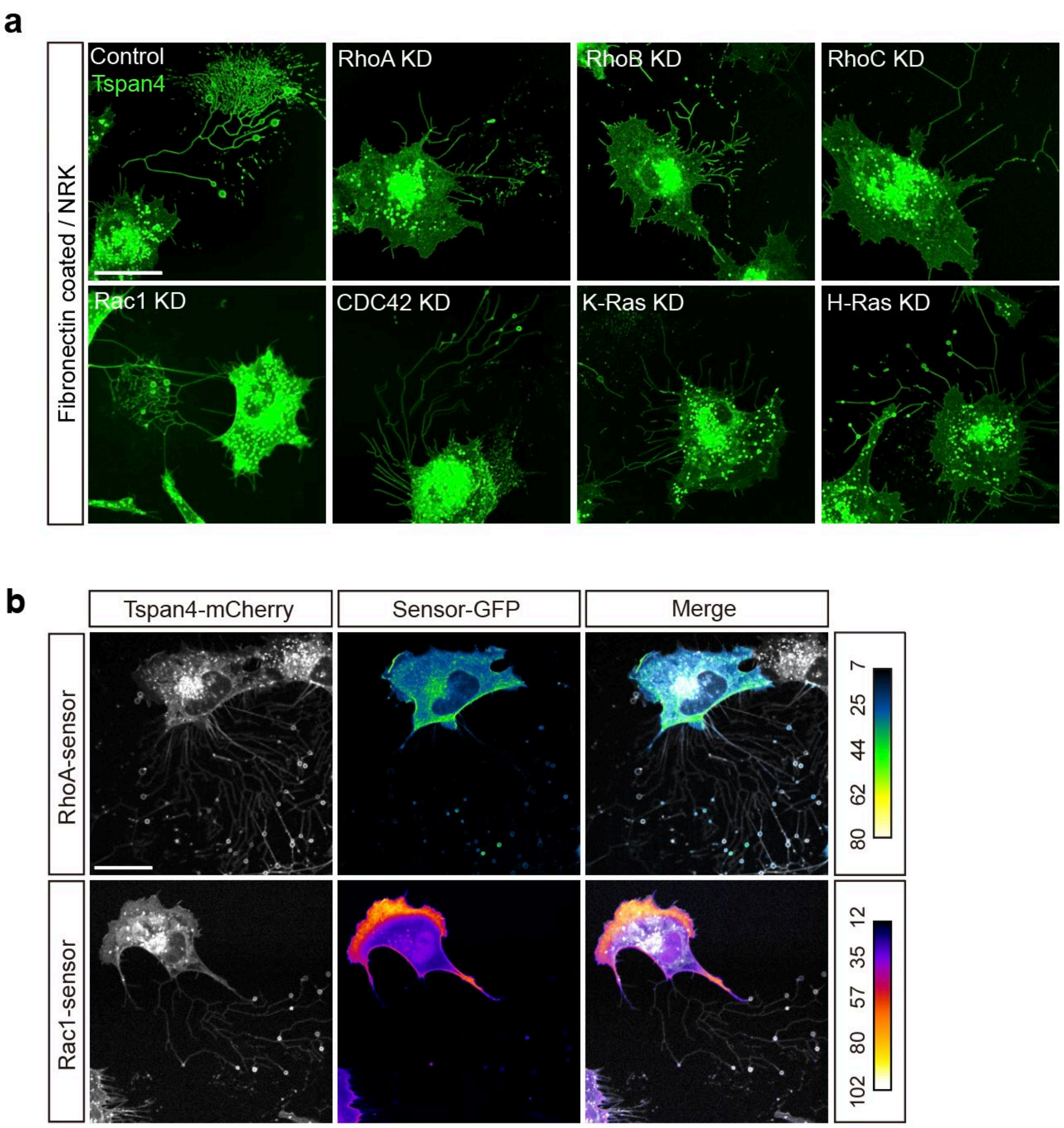

Supplementary Fig. S5 a Confocal images show altered migracytosis in some small GTPases knocked-down cell lines. TSPAN4-GFP NRK cells were seeded in fibronectin-pretreated plates and induced by Doxycycline (1 μg/mL) for 24 hours before plating. Scale bar, 25 μm. **b** Confocal images of NRK cells stably expressing TSPAN4-mCherry and RhoA-GFP or Rac1-GFP. Scale bar, 25 μm.

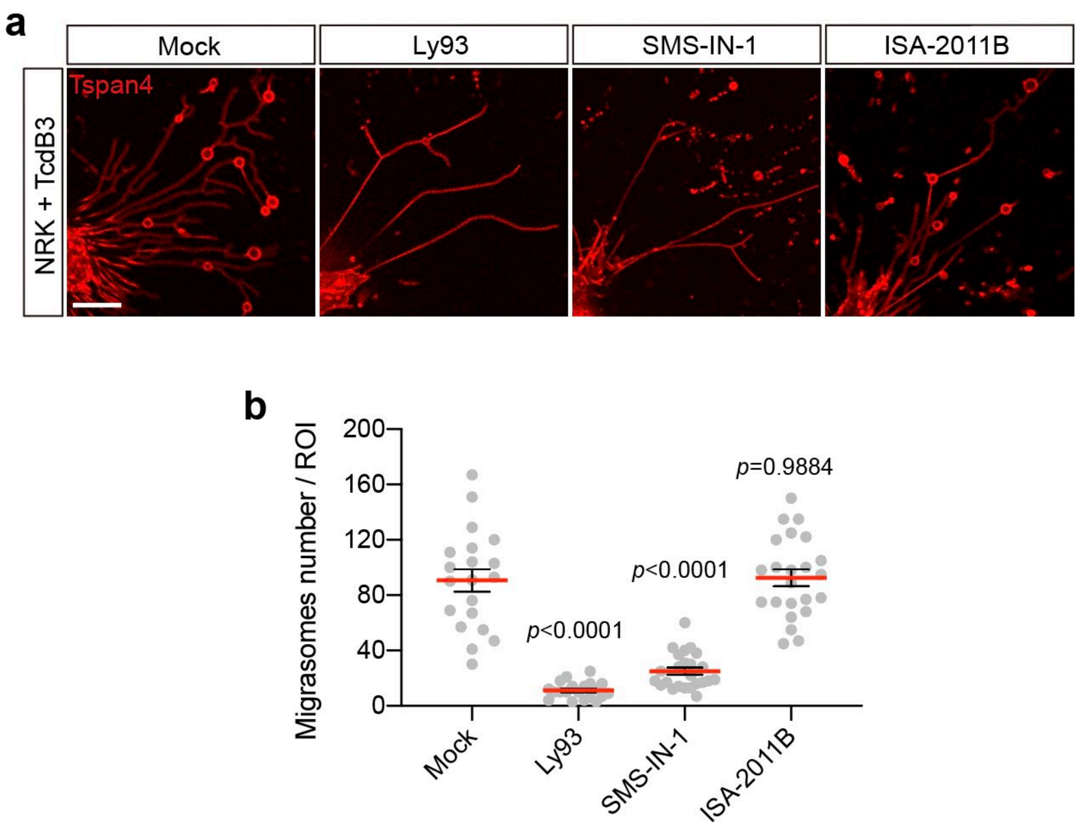

Supplementary Fig. S6 TcdB3-induced migracytosis requires SMS2 signaling but is independent of the activity of PIP5K1A. a Confocal images of NRK Tspan4-mCherry cells pretreated with ISA-2011B (20 μM), Ly93 (35 μM), or SMS2-IN-1 (30 μM) for 30 min, then followed by exposure to TcdB3 (0.2 pM). Scale bar, 10 μm. **b** Quantification of migrasomes per field from **a**. Data are presented as mean ± SEM; n = 20. *p*-values were calculated using ordinary one-way ANOVA followed by Dunnett's test.

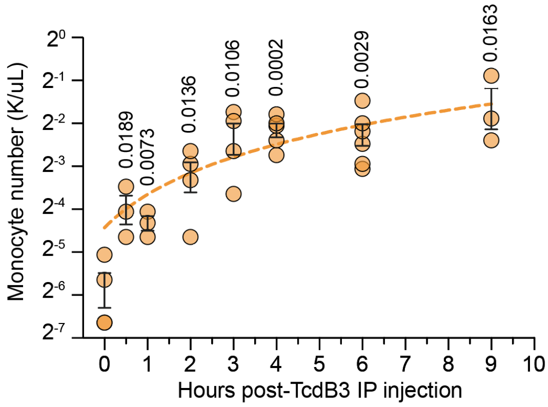

Supplementary Fig. S7 A surge of monocytes in the mouse blood appears after TcdB3 treatment. The numbers of blood monocytes at different time points post-IP injection of TcdB3 were shown. n = 3~6. *p* values were calculated using a two-tailed Mann-Whitney test.

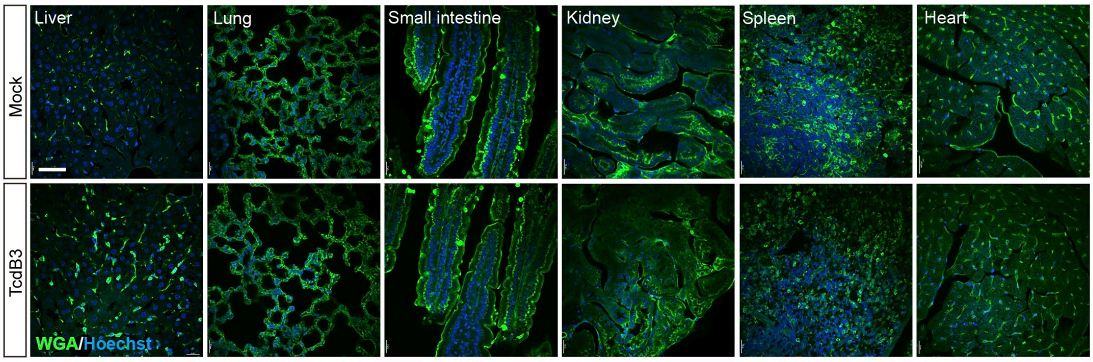

Supplementary Fig. S8 Confocal images depicting WGA signal variation between TcdB3 induction or not in various tissues. Mice were IP injected with TcdB3 (0.4 μg/kg) or saline for 6 hours. Their liver, lung, small intestine, kidney, spleen, and heart tissues were dissected, embedded, cut into sections, and stained by WGA (green) and Hoechst (blue). Scale bar, 50 μm.

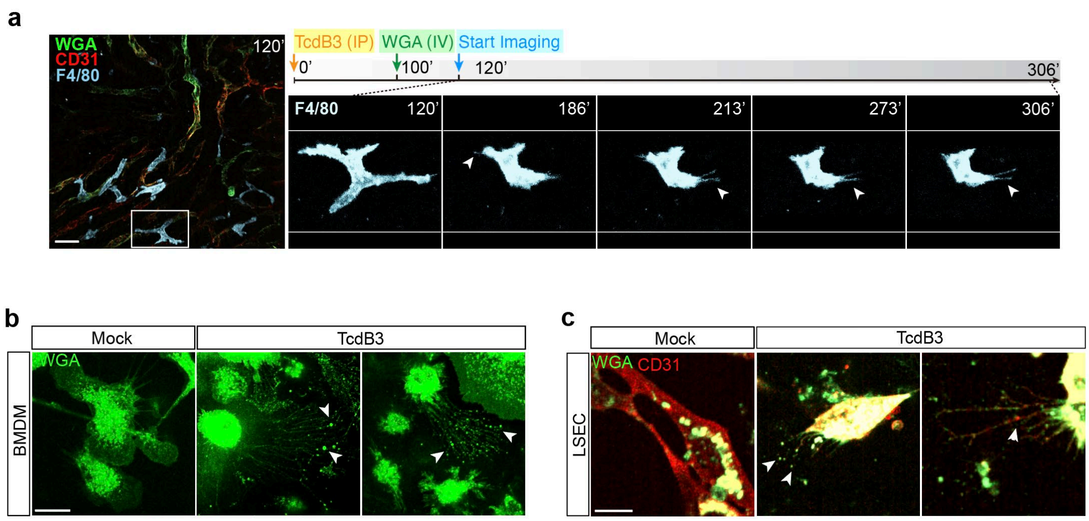

Supplementary Fig. S9 a Representative intravital images of the mouse liver after IP injecting of TcdB3 over time. CD31 (red) and F4/80 (Cyan) were labeled to indicate the LSECs and KCs in mouse liver. Scale bar, 30 μm. Time points post-toxin injection were indicated. **b** Representative confocal images show the primary bone marrow-derived macrophages (BMDM) stained by WGA-AF488. Scale bar, 10 μm. **c** Representative confocal images show the primary LSEC stained with WGA-AF488 (green) and an anti-CD31 antibody (red). Scale bar, 10 μm. Arrowheads indicate observed migrasome structures.

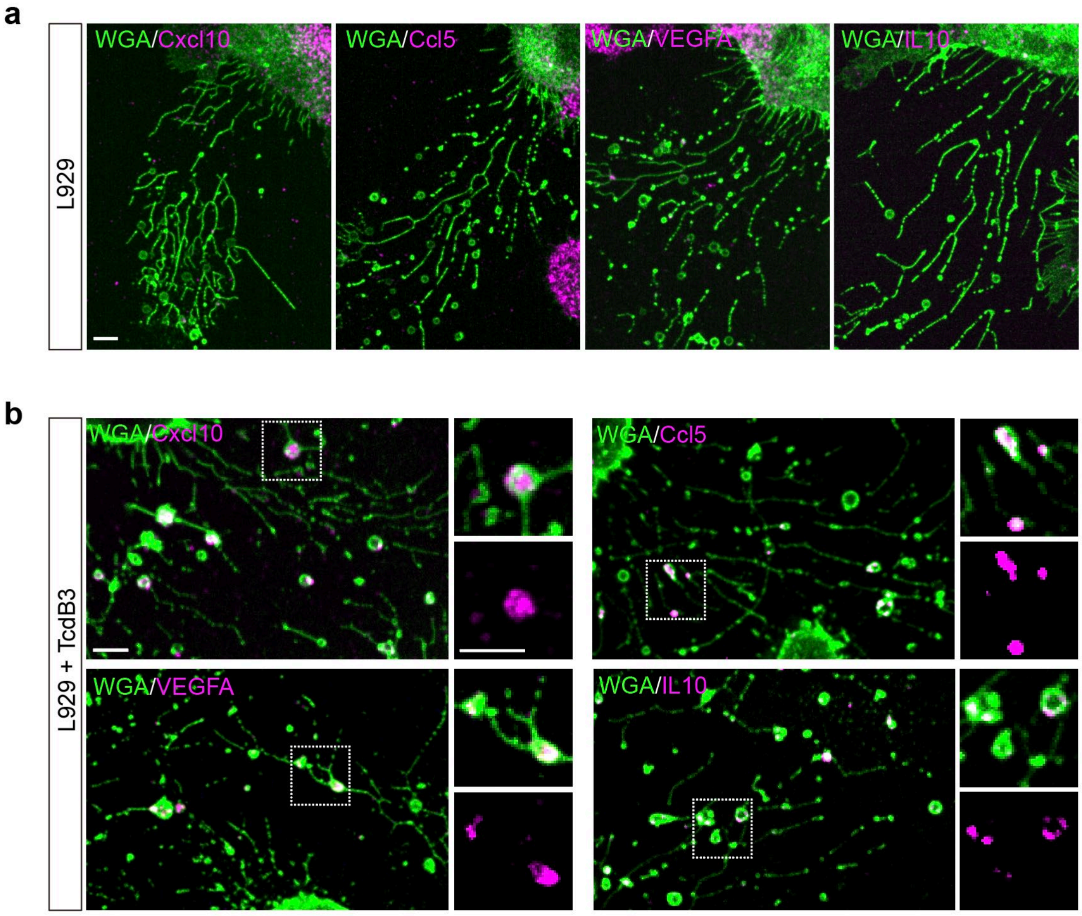

Supplementary Fig. S10 Confocal images reveal various cytokines and chemokines in the toxin-induced migrasomes. L929 cells were treated with 0.2 pM TcdB3 or not for 3 hours. Cells were stained with WGA-AF488 (green) and respective antibodies (magenta). Scale bar, 5 µm.

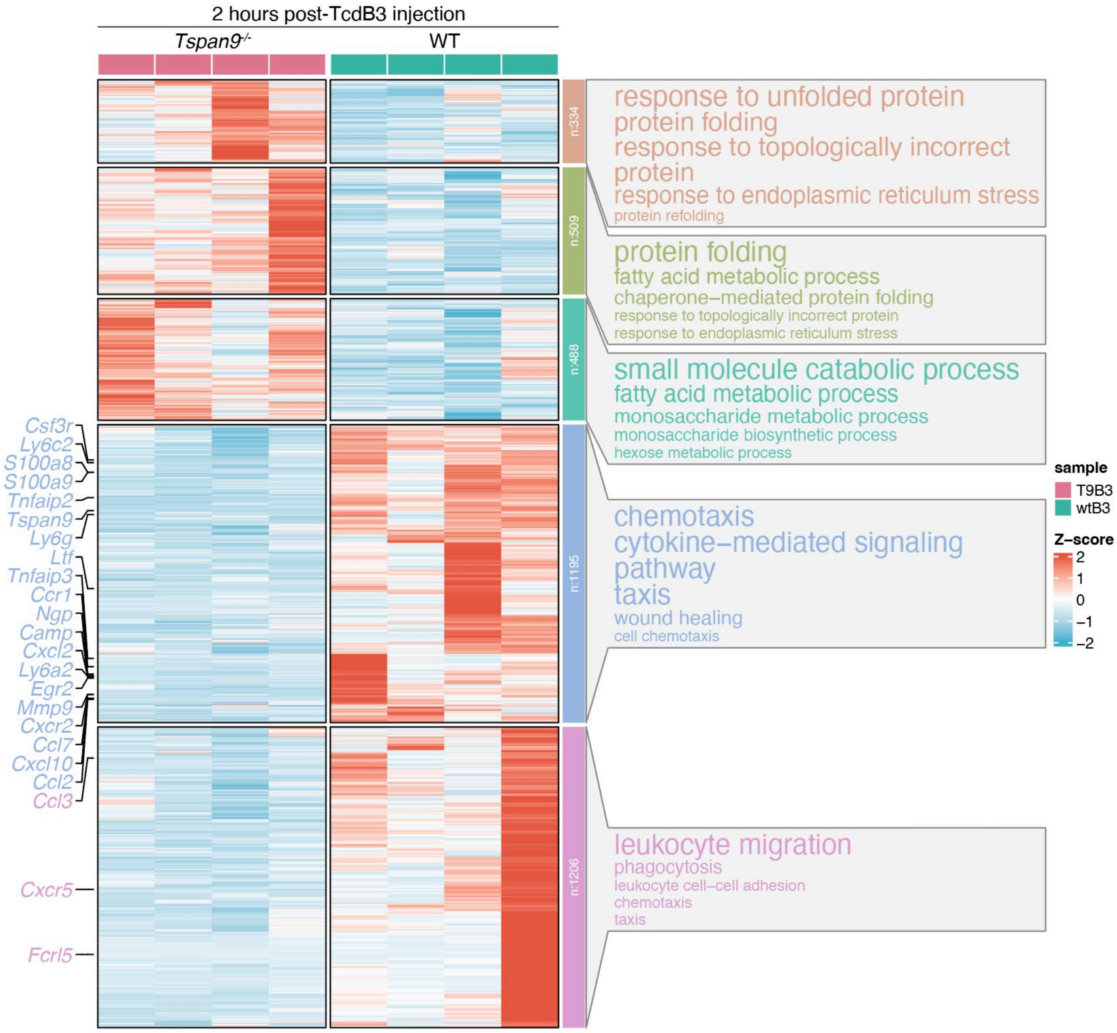

Supplementary Fig. S11 Heat map of gene expression profiles of liver tissues from the WT or *Tspan9*$^{-/-}$ mice. Tissues were collected 2 hours after IP injection of TcdB3 (0.4 μg/kg). Rows correspond to genes, and columns represent individual samples. The color gradient represents the scaled abundance levels, with warmer colors indicating higher abundance.

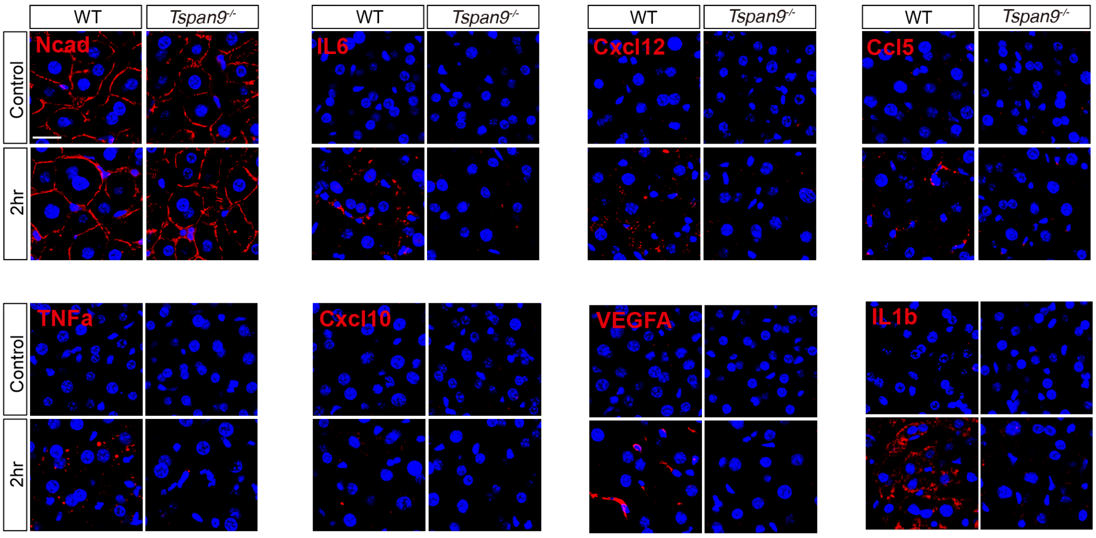

Supplementary Fig. S12 Confocal images reveal varied expression of several cytokines or chemokines in liver tissues from the WT or *Tspan9*$^{-/-}$ mice. Tissues were collected 2 hours after IP injection of TcdB3 (0.4 µg/kg) or saline, and stained with Hoechst (blue) and respective antibodies (red). Scale bar, 20 µm.

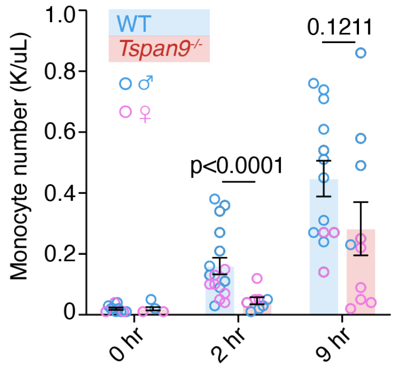

Supplementary Fig. S13 The blood monocyte numbers were measured 2 hours or 9 hours post-TcdB3 IP injection. Male (blue) and female (magenta) mice were labeled with different colors. n = 3~10. *p* values were calculated using two-way ANOVA Bonferroni's test.

Supplementary Video S1 | TcdB3 induced migrasome formation. Formation of TcdB3-induced migracytosis. Tspan4-GFP-expressing NRK cells were treated with 0.2 pM TcdB3 and time-lapse images were acquired using a NIKON spinning disk confocal microscope. Images were captured every 5 min for 795 mins. Related to Fig. 1f. (MP4 24945 kb)

Supplementary Video S2 | Migrasome formation *in vivo*. Intravital imaging of TcdB3 induced migracytosis in mouse liver. One hour after IP injecting of TcdB3 (0.6 μg/kg), time-lapse images of the mouse liver were captured using a Dragonfly 200 spinning disk confocal microscope. CD31-PE labels LSECs (red), WGA-AF488 labels WGA^{high} cells (green), and F4/80-APC labels KCs (cyan) in mouse liver. Images were captured every 3 min for 147 mins. Related to Fig. 4c. (MP4 7524 kb)
